# Schwann cell-derived Apolipoprotein D controls the dynamics of post-injury myelin recognition and degradation

**DOI:** 10.3389/fncel.2014.00374

**Published:** 2014-11-11

**Authors:** Nadia García-Mateo, Maria D. Ganfornina, Olimpio Montero, Miguel A. Gijón, Robert C. Murphy, Diego Sanchez

**Affiliations:** ^1^Lazarillo Lab, Departamento de Bioquímica y Biología Molecular y Fisiología, Instituto de Biología y Genética Molecular, Universidad de Valladolid-CSICValladolid, Spain; ^2^Mass Spectrometry Unit, Center for Biotechnology Development (CDB), Consejo Superior de Investigaciones CientíficasValladolid, Spain; ^3^Department of Pharmacology, University of Colorado DenverAurora, CO, USA

**Keywords:** Lipocalin, sciatic nerve, myelin phagocytosis, myelin degradation, macrophage, arachidonic acid, lysophosphatidylcholine

## Abstract

Management of lipids, particularly signaling lipids that control neuroinflammation, is crucial for the regeneration capability of a damaged nervous system. Knowledge of pro- and anti-inflammatory signals after nervous system injury is extensive, most of them being proteins acting through well-known receptors and intracellular cascades. However, the role of lipid binding extracellular proteins able to modify the fate of lipids released after injury is not well understood. Apolipoprotein D (ApoD) is an extracellular lipid binding protein of the Lipocalin family induced upon nervous system injury. Our previous study shows that axon regeneration is delayed without ApoD, and suggests its participation in early events during Wallerian degeneration. Here we demonstrate that ApoD is expressed by myelinating and non-myelinating Schwann cells and is induced early upon nerve injury. We show that ApoD, known to bind arachidonic acid (AA), also interacts with lysophosphatidylcholine (LPC) *in vitro*. We use an *in vivo* model of nerve crush injury, a nerve explant injury model, and cultured macrophages exposed to purified myelin, to uncover that: (i) ApoD regulates denervated Schwann cell-macrophage signaling, dampening MCP1- and Tnf-dependent macrophage recruitment and activation upon injury; (ii) ApoD controls the over-expression of the phagocytosis activator Galectin-3 by infiltrated macrophages; (iii) ApoD controls the basal and injury-triggered levels of LPC and AA; (iv) ApoD modifies the dynamics of myelin-macrophage interaction, favoring the initiation of phagocytosis and promoting myelin degradation. Regulation of macrophage behavior by Schwann-derived ApoD is therefore a key mechanism conditioning nerve injury resolution. These results place ApoD as a lipid binding protein controlling the signals exchanged between glia, neurons and blood-borne cells during nerve recovery after injury, and open the possibility for a therapeutic use of ApoD as a regeneration-promoting agent.

## Introduction

A detailed knowledge of the cellular and molecular mechanisms underlying the degeneration and restoration processes following an injury is fundamental to understand a set of basic physiological processes in the nervous system, as well as the regenerative potential of neurons suffering a degenerative condition. A wealth of information has been gathered in the last two decades concerning factors impacting on neuronal survival and axon regeneration following diverse forms of injury in the central (CNS) and peripheral nervous system (PNS) (reviewed by Vargas and Barres, 2007; Huebner and Strittmatter, 2009; Gaudet et al., 2011).

The degeneration and regeneration capabilities of the PNS have been deeply studied after Waller described the process in the XIX century (Koeppen, 2004). A number of molecular players involved in all steps of the Wallerian degeneration-regeneration process (WDR) of the PNS (axon degeneration and growth inhibition, myelin breakdown and clearance, and ultimately axon regeneration and remyelination) have been studied thoroughly. However, many details of this complex process are still unknown, and studying the role of newly uncovered implicated proteins is essential to fully understand it.

The Lipocalin Apolipoprotein D (ApoD) was initially related to the WDR of the PNS due to its injury-dependent over-expression in the rat peripheral nerve (Boyles et al., 1990; Spreyer et al., 1990). Our long-standing interest in this protein and its insect homologs led us to study the role of ApoD using genetically modified mouse models and a sciatic nerve crush paradigm (Ganfornina et al., 2010). By studying WDR at 14 and 48 days post-injury in genetically-modified mice with altered expression levels of ApoD, we uncovered affected cellular and molecular processes that contradicted the initial working hypothesis proposing that ApoD was implicated in remyelination, a late process that occurs after axon regeneration is permitted. Our results demonstrated earlier roles for ApoD facilitating myelin clearance, extracellular matrix remodeling and, later on, axon regeneration, but the molecular processes controlled by ApoD still remain unknown.

In this work we study an early stage of WDR, 7 days after a crush injury, in wild-type (WT) and ApoD-deficient (ApoD-KO) mice. At this time an injured nerve is at the mid-point of active axonal regrowth, and Schwann cells are switching into a distinct regeneration-promoting phenotype (Arthur-Farraj et al., 2012), thus representing a key point to analyze the early roles predicted for ApoD. In order to isolate the variables modified by ApoD we undertake three different levels of analysis: *in vivo*, using the sciatic nerve crush injury model; *ex vivo*, using cultured explants of sciatic nerves; and *in vitro* phagocytosis assays with primary macrophages fed with purified myelin. We also study the cellular source and localization of ApoD in response to injury.

## Materials and methods

### Animals, surgery, and injury procedures

Mice used in this study were age- and sex-matched littermates of two genotypes: Wild type (WT) and loss-of-function mutants for ApoD (ApoD-KO). ApoD-KO mice were generated by homologous recombination. The mutation is evidenced by PCR, in all experimental mice used, genotyping with two different primer pairs, as described previously (Ganfornina et al., 2008). In order to avoid potential maternal effects of ApoD, and to generate WT and ApoD-KO mice of homogenous genetic background, the experimental cohorts used in this study are the F1 generation of homozygous crosses of ApoD^−/−^ and ApoD^+/+^ littermates born from heterozygous crosses of an ApoD-KO line backcrossed for over 20 generations into the C57BL/6J background. Mice were maintained in positive pressure-ventilated racks at 25 ± 1°C with 12 h light/dark cycle, fed *ad libitum* with a standard rodent pellet diet (Global Diet 2014; Harlan Inc., Indianapolis, IN, USA), and allowed free access to filtered and UV-irradiated water.

Animals subjected to surgery and injury procedures (*n* = 21/genotype, 8 months old) were anesthetized via intraperitoneal injection with ketamine (100 mg/kg) and xylazine (10 mg/kg). Sciatic nerve crush was performed by compressing the nerve three times, 20 s each, with #5 forceps. Subsequently, muscles and skin were sutured and the animals were allowed to recover in a warmed cage. Sciatic nerves from both the injured and the control contralateral legs were removed 7 days post-crush injury (dPCI). A 2–3 mm fragment of the nerve distal to the lesion was stored at −80°C for RNA and protein expression analysis, and the remaining nerve was fixed and used in different experiments as explained below. Alternatively the nerve injury site fragment was stored at −80°C and used for lipid extraction.

Experimental procedures were approved by the University of Valladolid Animal Care and Use Committee and followed the regulations of the Care and the Use of Mammals in Research (European Commission Directive 86/609/CEE, Spanish Royal Decree 1201/2005).

### Immunohistochemistry

Nerve samples (*n* = 15 pairs/genotype) were fixed with 4% paraformaldehyde overnight at 4°C and embedded in paraffin following standard procedures. Longitudinal sections (4 μm) were performed with a rotary microtome (Microm), mounted in seven series on polysine slides (Menzel-Gläser), and dried.

Sections were dewaxed in xylene and rehydrated through an ethanol series into phosphate buffered saline (PBS). Before HRP immunohistochemisty, endogenous peroxidase was inactivated with H_2_O_2_ (0.9% in distilled H_2_O) for 5 min in the dark, followed by washes in PBS. Sections were then blocked and permeabilized with Triton X-100 (0.25% in PBS) and 1% normal goat serum (except for primary goat antibodies). The following antibodies were used: Rat monoclonal anti-Galectin 3 (American Type Culture Collection, ATCC); Rabbit serum anti-Mbp (Abcam); Goat polyclonal anti-Integrin αM (CD11b) (Santa Cruz Biotechnology); Goat polyclonal anti-ApoD (Santa Cruz Biotechnology); Mouse monoclonal anti-GFAP (Santa Cruz Biotechnology); HRP-conjugated Goat anti-Rat IgG (Abcam); HRP-conjugated Goat anti-Rabbit IgG and Goat anti-Mouse IgG (Dako); HRP-conjugated Donkey anti-Goat IgG (Santa Cruz Biotechnology). HRP development was performed with DAB (0.03%) and H_2_O_2_ (0.002% in 50 mM Tris, pH 8.0) in parallel for all sections probed with the same antibody. Sections were mounted with coverslips and Eukitt after dehydration and clearing with xylene.

For fluorescence immunohistochemistry, the following antibodies were used: Rat monoclonal anti-Galectin 3 (ATCC); Goat polyclonal anti-ApoD (Santa Cruz Biotechnology); Rabbit anti-S100 (Zymed); Mouse anti-GFAP (Santa Cruz Biotechnology); FITC-conjugated Goat anti-Rat, and Cy3.5-conjugated Goat anti-Rabbit (Abcam). After several washes in PBS, sections were mounted with Vectashield-DAPI (Vector Labs).

For Luxol Fast Blue (LFB) staining, sections were incubated in LFB solution at 37°C overnight, washed in 95% ethanol, differentiated in 0.005% lithium carbonate followed by 70% ethanol, and mounted after dehydration and clearing in Eukitt.

Sections were visualized and photographed with an Eclipse 90i (Nikon) fluorescence microscope equipped with a DS-Ri1 (Nikon) digital camera. Confocal images were obtained in a DMI 6000B microscope with TCS SP5 X confocal system and a WLL laser (Leica) controlled by LAS AF software (Leica).

### Quantitative RT-PCR

Distal portions of nerves (*n* = 6/genotype) or cells used for mRNA expression studies were stored at −80°C and RNA was extracted with RNeasy Lipid Tissue Mini Kit (Qiagen) using QIAzol Lysis Reagent (Qiagen). RNA concentration was measured with a Nanodrop spectrophotometer and quality assessed by agarose electrophoresis. Following DNAse treatment, 500 ng of total RNA was reverse-transcribed with PrimeScript (Takara Bio Inc., Otsu, Japan) according to the manufacturer instructions by using Oligo-dT primers and random hexamers. The resulting cDNA was used as template for quantitative real-time RT-PCR using SybrGreen (SYBR® Premix Ex Taq™ kit, Takara). The primers used for RT-qPCR are shown in Table 1. Rpl18 was used as the reference gene.

**Table 1:** **Primers used for quantitative real-time RT-PCR**.

**Primer name**	**Sequence**
Mouse Rpl18-Forward	5′-TTCCGTCTTTCCGGACCT
Mouse Rpl18-Reverse	5′-TCGGCTCATGAACAACCTCT
Mouse ApoD-Forward	5′-GAAGCCAAACAGAGCAACG
Mouse ApoD-Reverse	5′-TGTTTCTGGAGGGAGATAAGGA
Mouse Ccl2-Forward	5′-TCCCTGTCATGCTTCTGGGCCT
Mouse Ccl2-Reverse	5′-GCTTCTTTGGGACACCTGCTGCT
Mouse Tnf-Forward	5′-TCTTCTCATTCCTGCTTGTGGCAG
Mouse Tnf-Reverse	5′-TGGTTTGCTACGACGTGGGCT
Mouse Il1b-Forward	5′-TGTAATGAAAGACGGCACACCCAC
Mouse Il1b-Reverse	5′-GGCTTGTGCTCTGCTTGTGAGG
Mouse Il10-Forward	5′-CAGAGCCACATGCTCCTAGAGC
Mouse Il10-Reverse	5′-GGCCATGCTTCTCTGCCTGGG
Mouse Il6-Forward	5′-CACATGTTCTCTGGGAAATCGTGGA
Mouse Il6-Reverse	5′-CTCCAGGTAGCTATGGTACTCCAGAA
Mouse Emr1-Forward	5′-AAGCAGTGCAGGGCAGGGAT
Mouse Emr1-Reverse	5′-GCAAGATGGTGCCCAGAGTGGA
Mouse Cpla2-Forward	5′-GTGAGGGGCTTTATTCCACA
Mouse Cpla2-Reverse	5′-GGTGAGAGTACAAGGTTGACA
Mouse Mmp9-Forward	5′-TACACGGAGCACGGCAACGG
Mouse Mmp9-Reverse	5′-CGCACAGCTCTCCTGCCGAG

Transcription levels of mRNA were estimated with the ΔΔC_T_ method (Livak and Schmittgen, 2001). Transcripts were normalized to Rpl18 for each condition. Statistically significant differences of gene transcriptional changes were evaluated with a Mann-Whitney *U*-test (Yuan et al., 2006) using ΔC_T_ of each replica (calculated by subtracting the average CT of the reference gene for each sample). The statistical level of significance was set at *P* < 0.05.

### Immunoblot analysis

Peritoneal macrophages or distal fragments of each nerve (*n* = 8/genotype) were pooled by experimental condition and genotype, and proteins were extracted from the organic phase of QIAzol Lysis Reagent-treated samples after RNA extraction following the manufacturer's protocol. Total protein concentration was evaluated by bicinchoninic acid assay (MicroBCA, Pierce, Rockford, IL, USA).

Immunoblot analyses were performed with 10–20 μg of total protein/lane transferred to PVDF membranes using standard procedures and exposed to the following primary antibodies: Rat monoclonal anti-Galectin 3 (ATCC); Goat polyclonal anti-ApoD and Goat polyclonal anti-Integrin αM (CD11b) (Santa Cruz Biotechnology, Inc.); Rabbit polyclonal anti-Mbp (Abcam); Rabbit polyclonal anti cPLA_2_ alpha, and rabbit polyclonal anti-phospho cPLA_2_ alpha (Cell Signaling); Mouse anti-GFAP (Santa Cruz Biotechnology, INC). HRP-conjugated Goat anti-Rat, Goat anti-Mouse or Donkey anti-Goat IgG (Santa Cruz Biotechnology, Inc.) were used as secondary antibodies. HRP-conjugated anti-β actin antibody (Sigma, St Louis, MO, USA) was used to normalize protein loads. Membranes were developed with ECL reagents (Millipore, Billerica, MA, USA), and the signal visualized with a digital camera (VersaDoc, BioRad). The integrated optical density of the immunoreactive protein bands was measured in images taken within the linear range of the camera, avoiding signal saturation.

### Myelin isolation and labeling

Myelin was isolated from whole brain of 3 month-old mice by sucrose density-gradient centrifugation, according to the Norton and Poduslo method (1973). We used six independent myelin preparations per genotype (*n* = 3 brains/preparation). Myelin protein content was determined with MicroBCA Protein Assay (Pierce) and monitored by SDS-PAGE and Coomassie blue staining. Myelin (1 mg/ml) was fluorescently labeled by incubation with 12.5 mg/ml of the lipophilic dye 1,1″-dioctadecyl-3,3,3′,3′-tetramethylindocarbocyanide perchlorate (DiI; Sigma) for 30 min at 37°C. Excess of DiI was removed by washing with sterile PBS followed by centrifugation (20 min at 24000 *g*). Myelin fluorescence was determined in a Shimadzu RF-5301PC spectrofluorometer. Excitation wavelength was 545 nm. Fluorescence emission was recorded from 490 to 600 nm. Labeled myelin was stored in small aliquots at −20°C in the dark.

Myelin particle area and total counts were measured using ImageJ software (Rasband, W.S., ImageJ, U. S. National Institutes of Health, Bethesda, Maryland, USA, http://imagej.nih.gov/ij/, 1997–2014) and thresholded images of DiI-labeled myelin preparations freshly spread on microscope slides.

### Primary culture of peritoneal macrophages

Peritoneal macrophages were harvested in 5 ml cold PBS, either as naïve cells or 4 days after an intraperitoneal injection of 2.5 ml of 3% thioglycollate (TG) (Difco, USA) in 4–8 month-old mice (*n* = 6/genotype). The cells were subsequently washed in PBS and cultured in Dulbecco's Modified Eagle's medium (DMEM) with 10% heat-inactivated fetal bovine serum (FBS), 1% L-Glutamine and 1% P/S/A solution (Penicillin 10 U/μL, Streptomycin 10 μg/μL, Amphotericin B 25 μg/mL), henceforth called “complete medium.”

### Myelin phagocytosis assay

Macrophages were plated in 24-well tissue culture plates (7.5 × 10^5^ cells/well) in the presence of complete medium for 5 h. Non-adherent cells were removed by washing twice with PBS, and 1 ml of complete medium was added. Over 95% of the cultured cells were macrophages as determined by immunofluorescence with macrophage markers. The cells were incubated with 5 μg of DiI-labeled myelin (10 μg/ml) for 90 min at 37°C in 5% CO_2_. After the incubation period, non-phagocytosed and unbound myelin was removed by washing twice with PBS. Cells were detached by incubation at 37°C in 5% CO_2_for 10 min with Trypsin/EDTA (Lonza) and using a cell scraper. After resuspension in PBS, the amount of myelin phagocytosed was determined by measuring cellular fluorescence intensity recorded in the FL2 channel (560–590 nm) of a Gallios Flow Cytometer (Beckman Coulter). The histograms of control macrophages without myelin at each time point were used to define the fluorescence threshold value to consider events as DiI positive macrophages. The percentage of positive cells was used as a measure for binding and uptake of the DiI-labeled myelin. The percentage of myelin-positive macrophages was plotted against time of myelin exposure, and the rate of change of this variable was calculated as the slope of the curve obtained between 20 and 60 min. Phagocytosis analyses were performed in 3–4 independent experiments, with 3 macrophage-myelin samples/experiment).

Fluorescence microscopy was used to visualize myelin uptake by macrophages. For this purpose, 2.5 × 10^5^ cells were cultured on 12 mm coverslips and 5 μg of DiI-labeled myelin (10 μg/ml) was added per well in complete medium. Cells were incubated at 37°C in 5% CO_2_. Following incubation and extensive washout of non-phagocytosed myelin particles with PBS, cells were fixed in 4% formaldehyde in PBS, washed in PBS and mounted with Vectashield-DAPI (Vector Labs). Labeled cells were visualized with an Eclipse 90i (Nikon) fluorescence microscope equipped with a DS-Ri1 (Nikon) digital camera. The area and total counts of myelin particles ingested by cultured macrophages in 60 min were measured from thresholded images with ImageJ software.

For the exogenous addition of ApoD, human ApoD purified from breast cystic fluid (Ruiz et al., 2013) was added at different concentrations (10–100 nM) to the myelin preparation 5 min before its addition to the macrophage culture.

### Sciatic nerve explants model for *ex vivo* analysis of Wallerian degeneration

To study axonal degeneration *ex vivo*, sciatic nerves were dissected from adult WT and ApoD-KO mice (*n* = 3/genotype), and their epineurium was removed. Nerve segments of ~5 mm were incubated on a Nucleopore Track-Etch Membrane (Whatman) in 4-well dishes containing 500 μL of DMEM with 10% heat-inactivated FBS, 1% L-Glutamine and 1% P/S solution (Penicillin 10 U/μL, Streptomycin 10 μg/μL,). Explants were cultured at 37°C and 5% CO_2_ for 7 days, with a change of medium every 2 days. At day 7, the medium was removed and the nerves were washed with PBS before freezing them at −80°C.

### Mass spectrometry analysis of lipids from injured nerves and from myelin

Lipids from intact and injured nerves (*n* = 6/genotype) were extracted using a modified Bligh and Dyer (1959) method, using 0.1 N HCl instead of water. The extracted lipids were resuspended in 150 μL of methanol:water (9:1, v/v) and kept at −20°C until analysis. Protein concentration in the extracted samples was estimated by MicroBCAanalysis (Pierce). Ultraperformance liquid chromatography interfaced to a time-of-flight mass spectrometer (UPLC-QToF-MS) was performed using an Acquity UPLC System and SYNAPT HDMS G2 (Waters, Milford, USA). A two solvent gradient elution was used for compound separation on an Acquity BEH C18 column (2.1 × 50 mm, 1.7 μm particle size, temperature 30°C), as follows: (i) initial, 80% A; (ii) 0–1 min, 80% A; (iii) 1.0–5.5 min, 100% B; (iv) 5.5–7.5 min, 100% B; and (v) 7.5–10.0 min: 80% A. Solvent A consisted of methanol/water/formic acid (50:50:0.5, v/v/v) with 5 mM ammonium formate, and solvent B contained methanol/acetonitrile/formic acid (59:40:0.5, v/v/v) with 5 mM ammonium formate. The flow rate used was 0.5 mL/min, and the injection volume was 7.5 μL. Compounds were detected as positive ions using a MS^E^ method with low (full scan) and high (fragmentation at 20–30 V) energy functions. MS parameters were: capillary, 0.9 V; sample cone, 18 V; source temperature, 90°C; desolvation temperature, 450°C; cone gas, 20 L/h; desolvation gas, 900 L/h. Analysis in negative mode was also conducted in order to obtain data on the acyl chains esterifying the glycerol backbone. Compounds were identified by *m/z* search within ± 0.01 Da in the LIPIDMAPS database (www.lipidmaps.org) and coincidence of the elemental formula composition. Specific fragments obtained with the high energy function were used to confirm phospholipid class identification (*m/z* 184.07 for PCs in positive-ion mode; *m/z* 196.01 for PEs and *m/z* 223.03 or *m/z* 241.02 for PIs in negative-ion mode). The areas of the chromatographic peaks obtained in the extracted ion chromatogram (EIC) from the full scan (low-energy function) were determined by integration using the MassLynx software tool.

Arachidonic acid (AA) and lysophosphatidylcholines (LPC) were analyzed in lipid extracts from pools of 6 control and 6 injured (injury site, 7d-PCI) fragments (≈1 mm^3^) per genotype. AA was measured in negative-ion mode using the UPLC-MS method described in Municio et al. (2013), whereas LPCs were measured in positive-ion mode as indicated above. Total LPC normalized to protein content are represented as percentage of WT control nerves. The AA concentration in extracts was calculated after a linear regression of peak area to concentration was determined using an AA external standard (Sigma-Aldrich, A9673). AA concentration normalized to protein content is plotted.

Whole brain myelin was extracted from 6 to 7 pools (2 brains each) per genotype. Independent lipid extracts were prepared from each pool as explained above, and deuterated internal standards (Avanti Polar Lipids) for each of six major phospholipid classes were added (25 ng each): [^2^H_31_]16:0/18:1-PA, [^2^H_31_]16:0/18:1-PC, [^2^H_31_]16:0/18:1-PE, [^2^H_31_]16:0/18:1-PG, [^2^H_31_]16:0/18:1-PI and [^2^H_31_]16:0/18:1-PS. Samples were analyzed by LC/MS/MS using an API3200 triple quadrupole mass spectrometer (AB SCIEX) connected to an HPLC dual pump system (Shimadzu). Samples were dried under a stream of dry nitrogen gas and dissolved in 100 μL of a mixture of 75% HPLC solvent C (hexanes/isopropanol 30:40, v/v) and 25% solvent D (5 mM ammonium acetate in hexanes/isopropanol/water 30:40:7, v/v/v). Normal-phase chromatography was performed using a silica column (Ascentis, 150 × 2.1 mm, 5 μm particle size, Supelco) at a flow rate of 200 μL/min. Solvent D was maintained at 25% for 5 min, increased gradually to 60% in 10 min and then to 95% in 5 min, and was held for 20 min before re-equilibration for 15 min. Analysis was performed in the negative-ion mode using multiple-reaction monitoring (MRM) of different molecular species containing combinations of the more common fatty acyl chains (Steinhauer et al., 2009). The precursor ions monitored were the molecular ions [M-H]^−^, except for PC in which case the acetate adducts [M+CH_3_COO]^−^ were monitored. The product ions analyzed after collision-induced decomposition were the carboxylate anions corresponding to the acyl chains. Results are reported as the ratio between the integrated area of each analyte and the integrated area of the corresponding internal standard. Since no standard dilution curves were used, these results cannot be used to quantify absolute amounts of the analytes, but they are useful to identify potential changes across the samples of different genotypes.

### Ligand binding assays by tryptophan fluorescence titration

Ligand binding to ApoD was performed as previously described (Sanchez et al., 2008; Ruiz et al., 2013). Fluorescence measurements were conducted with a Shimadzu RF-5301PC spectrofluorometer with a quartz cuvette (Hellma 105.251-QS, 3 mm path length). Temperature was held at 22 ± 0.1°C. The excitation wavelength was 295 nm (selective for tryptophan residues). Emission was recorded at the 340 nm peak with slit width set at 5 nm. The fluorescence value was used to calculate the apparent equilibrium dissociation constant (*K_D_*.) Data were analyzed using the RFPC software package (Shimadzu). Human ApoD from cyst fluid was diluted to a concentration of 0.5 μM in 10 mM phosphate buffer, 150 mM NaCl, 1 mM EDTA, pH 7.0, so that the starting intrinsic fluorescence of the protein lies within the dynamic range of measurement. A 100 mM stock of LPC (Sigma-Aldrich, L1381) was prepared in ethanol. Protein solutions (100 μl) were titrated with either four or eight additions of ligand, adding each time 1 or 0.5 μl aliquots of a 1:1000 dilution of ligand in phosphate buffer. This dilution of ligand stock into the aqueous solution was made immediately before addition to the protein. After addition of each ligand aliquot, the mixture was equilibrated for 2 min in the dark before fluorescence was recorded.

The fluorescence spectrum in the presence of ligand was subtracted from a carrier baseline obtained by titration of the protein with the same amounts of carrier without ligand. Ethanol final concentration was ≤0.004%. The corrected fluorescence at 340 nm vs. the total concentration was fitted as previously described (Sanchez et al., 2008). *K_D_* was calculated under the assumption of a single binding site, consistent with known Lipocalin structural properties. A tryptophan residue conserved along the entire Lipocalin family is located at the binding pocket surface, and is predicted to be the residue responsible for most of the intrinsic fluorescence changes observed.

### Statistical analyses

Statistical analyses were performed using Sigmaplot (v11.0) and Statgraphics Plus (v5.0). A *P*-value of 0.05 was considered a threshold for statistical significance.

## Results

Our previous work studying WDR in the sciatic nerve of mice lacking or over-expressing ApoD (Ganfornina et al., 2010) uncovered an important function for ApoD in the clearance of myelin preceding axon regeneration. Myelin clearance is performed both by resident Schwann cells (SCs), which transform their phenotype into a phagocytic repair cell, and by invading macrophages from the bloodstream. Thus, we set up to study the effects of changing ApoD expression levels on the WDR process 7 days after crush injury (7d-PCI). This *in vivo* sciatic nerve study is combined with *in vitro* phagocytosis assays in isolated primary macrophages and an *ex vivo* nerve injury model, to better dissect the molecular elements involved using experimental systems with different degrees of cellular complexity.

### ApoD expression in glial cells in the intact and injured nerve at 7d-PCI

**Figure 1:**
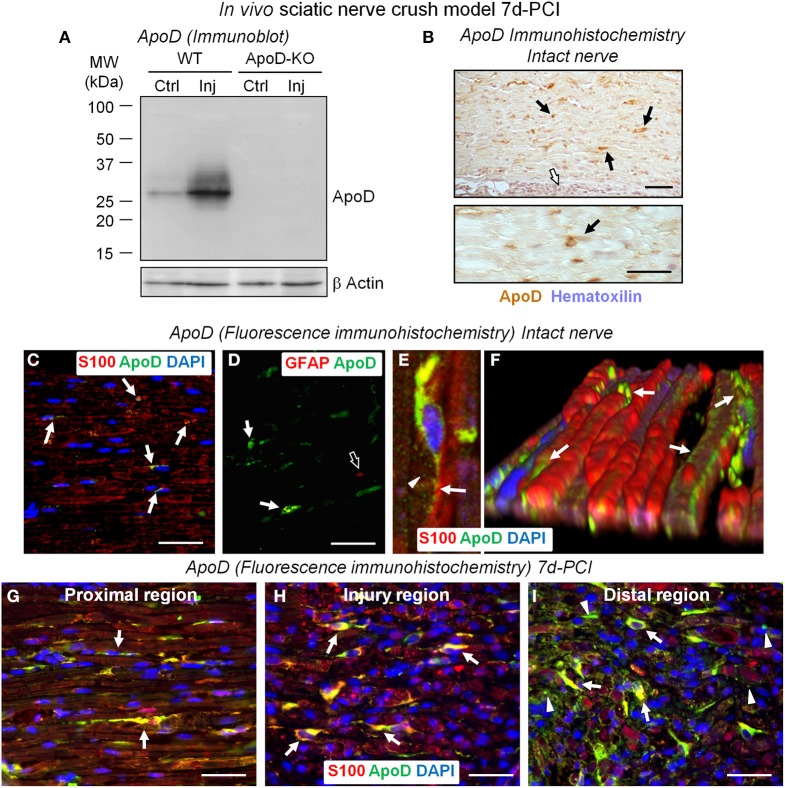
**ApoD is over-expressed by Schwann cells at 7 days after sciatic nerve crush injury**. **(A)** Immunoblot analysis of ApoD in the region distal to the nerve lesion of WT and ApoD-KO mice. High levels of ApoD protein are observed 7d-PCI. **(B)** HRP-immunohistochemistry of ApoD in sagittal sections of intact sciatic nerves. Scattered ApoD-positive cells are seen in the endoneurium (arrows), while no labeling is seen in the perineurium (white arrow). **(C–F)** Fluorescence-immunohistochemical detection of ApoD in sagittal sections of intact sciatic nerves. Co-localization of ApoD and S100 (arrows in **C**) confirms Schwann cells (SCs) as a source of ApoD in sciatic nerves. A subset of non-myelinating SCs also expresses ApoD as evidenced by co-localization with the GFAP marker (arrows in **D**) compared to GFAP-only signal (open arrow in **D**). Confocal image **(E)** and a 3-D reconstruction of a Z-stack of confocal images **(F)** of fluorescence immunohistochemistry show ApoD labeling in secretion vesicles (arrowhead in **E**) and the SCs outer sheath (arrows in **E,F**). **(G–I)** Co-localization of ApoD and the SC marker S100 in sagittal sections of a crushed WT sciatic nerve 7d-PCI at different positions with respect to the injury site. Arrows point to ApoD labeling in intact and denervated SCs. Arrowheads in **(I)** (region distal to the crush) show ApoD adjacent to S100-positive myelin debris. Calibration bars: 50 μm.

Before assaying the role of ApoD in the early myelin clearance and macrophage recruitment stage of nerve injury, we first confirmed the induction of ApoD expression in the distal region of crushed nerves at 7d-PCI by immunoblot (Figure 1A). ApoD protein over-expression had been described in the rat at 21 days after denervation (Boyles et al., 1990), but no data was available from mice.

Knowledge of the cellular localization of ApoD is also necessary in order to show its source. In the intact nerve, ApoD immunolocalizes in cells of elongated nuclei with labeling located along the sheaths of myelinated axons (arrows in Figure 1B). In contrast, ApoD is absent in perineurial cells (white arrow in Figure 1B). Cell types expressing ApoD were confirmed by co-localization with the myelinating SC marker S100 and non-myelinating SC marker GFAP. ApoD is observed in a subset of myelinating (Figure 1C) and non-myelinating SCs (Figure 1D), suggesting a dynamic spatiotemporal regulation of its expression. Confocal microscopy and 3D-reconstructions of Z-stacks show ApoD located in secretion vesicles (arrowhead in Figure 1E) and the outer sheath of SCs (arrows in Figures 1E,F).

In injured nerves, and consistent with our immunoblot results (Figure 1A), the number of ApoD-positive cells increases at 7d-PCI, showing extensive co-localization with S100 (arrows in Figures 1G–I). We can identify ApoD-expressing cells as S100-positive SCs that are known to be undergoing a phenotypic switch toward a distinct repair-focused cell at this stage after injury (Arthur-Farraj et al., 2012). These cells are involved in the process of myelin phagocytosis and are providing signals to recruit hematogenous macrophages for *en masse* phagocytosis. Interestingly, ApoD is also observed in S100-positive myelin debris in the region distal to the injury (arrowheads in Figure 1I). This finding is consistent with the detection of ApoD in myelin preparations (**Figure 6D**, and Suresh et al., 1998; Jahn et al., 2009).

The observed expression supports the hypothesis that glia-derived, myelin-associated ApoD plays a role in injured nerves at times when SCs are switching phenotypes and myelin phagocytosis and macrophage recruitment are taking place.

### Alterations in Wallerian degeneration in the absence of ApoD at 7d-PCI

#### Cytokine-chemokine network response

**Figure 2:**
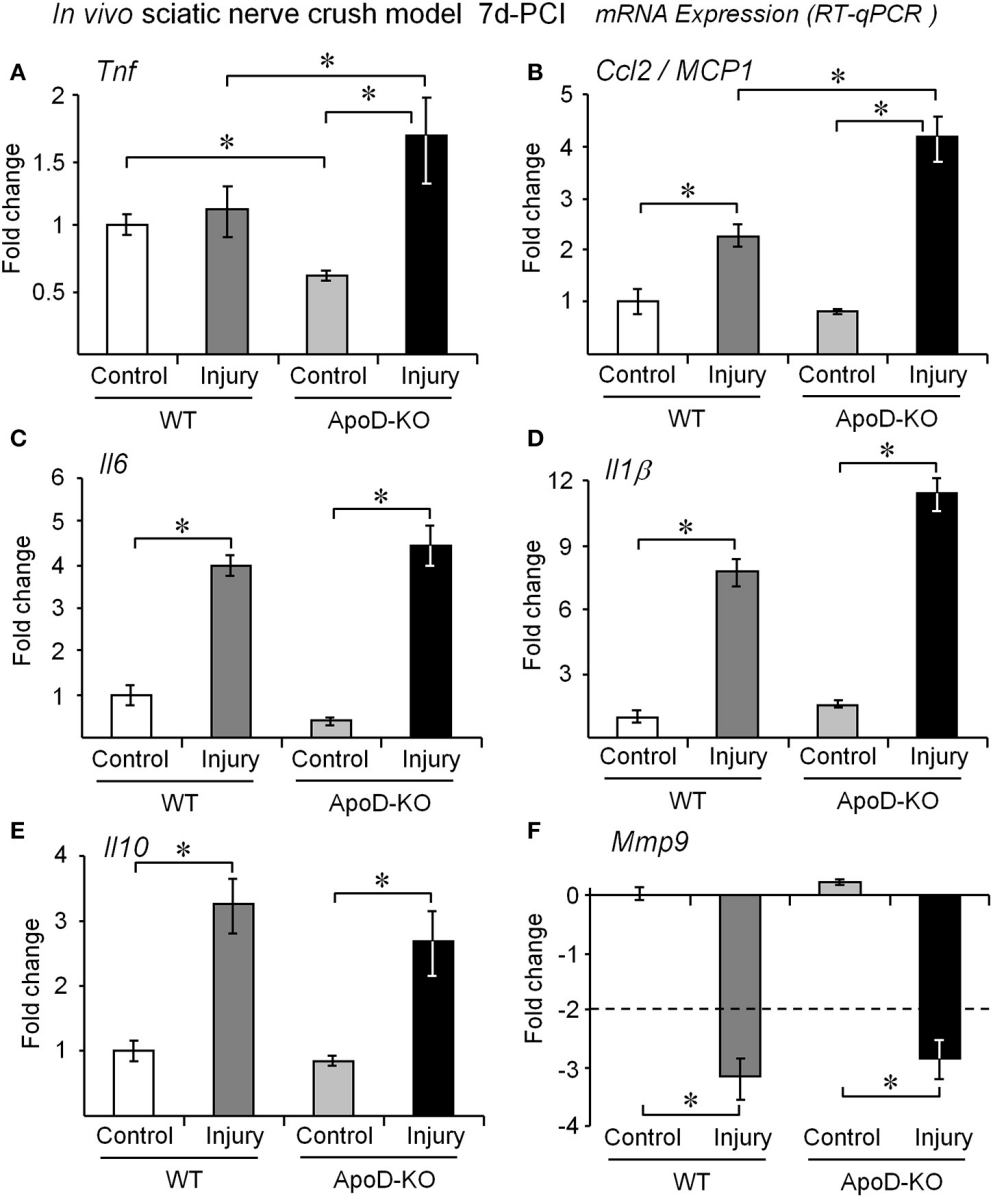
**ApoD alters the expression levels of genes involved in macrophage recruitment *in vivo***. Quantitative RT-PCR analyses of **(A)** Tnf, **(B)** Ccl2/MCP1, **(C)** Il6, **(D)** Il1β, **(E)** Il10, and **(F)** Mmp9 expression. The mRNA was extracted from the region distal to the lesion of WT and ApoD-KO mice at 7d-PCI. The absence of ApoD results in exacerbated Ccl2/MCP1 and Tnf signaling in the injured nerve. The transcriptional responses of Il1β, Il6, Il10, and Mmp9 are independent of ApoD. Statistical differences were assayed by Mann-Whitney *U*-test. Asterisks show statistically significant differences (*P* < 0.05).

We first monitored the transcriptional response to injury of several cytokines, chemokines and other molecules related to WDR in WT and ApoD-KO nerves. At 7d-PCI, the mRNA expression of the cytokine Tnf is similar to the basal level in WT nerves, as expected after its early induction peak (Kleinschnitz et al., 2004; Iwatsuki et al., 2013). However, Tnf mRNA is found significantly increased in the 7d-PCI ApoD-KO nerves (Figure 2A), suggesting a sustained expression. Since Tnf expression is down-regulated in the ApoD-KO intact nerve, injury-dependent up-regulation of Tnf is enhanced in the absence of ApoD. An over-response to the injury situation also occurs in ApoD-KO mice for the chemokine and macrophage chemoattractant protein MCP1, which is also induced at 7d-PCI in the distal part of the injured nerve (Figure 2B), and its induction is enhanced in the absence of ApoD. However, at this time after injury we did not observe significant ApoD-dependent differences for the response of the pro-inflammatory cytokines Il6 and Il1β (Figures 2C,D), the anti-inflammatory cytokine Il10 (Figure 2E), or the matrix metalloproteinase Mmp9 (Figure 2F). Thus ApoD-KO injured nerves show alterations in a particular subset of the cytokine-chemokine network organizing myelin clearance. This results in an altered inflammatory state that might result from, and influence, the activity of transdifferentiated Schwann cells or the recruitment and activation of blood-borne monocytes.

#### Cellular response

**Figure 3:**
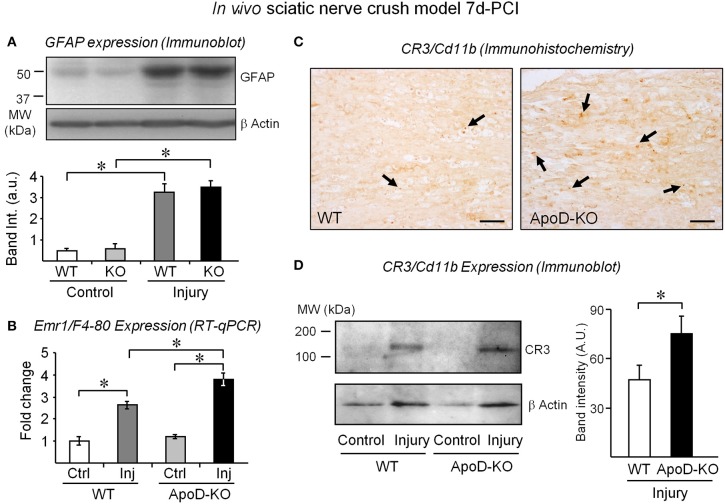
**The expression of macrophage markers is exacerbated in injured nerves in the absence of ApoD**. GFAP protein expression normalized to β-actin **(A)** and Emr1/F4-80 mRNA expression **(B)** in intact nerves and in the distal region of injured nerves of WT and ApoD-KO mice at 7d-PCI. **(C)** Representative examples of CR3/Cd11b immunohistochemistry in sagittal sections of the injury region of crushed sciatic nerves. Arrows point to CR3-positive macrophages. Calibration bars: 50 μm. **(D)** Immunoblot analysis of the CR3/Cd11b protein in the region distal to the lesion of WT and ApoD-KO mice at 7d-PCI. Statistical differences were assayed by Student's *t*-Test **(A,D)** and Mann-Whitney *U*-test **(B)**. Asterisks show statistically significant differences (*P* < 0.05).

To identify which cellular responses are affected by the absence of ApoD, we first estimated the amounts of non-myelinating Schwann cells by measuring the expression of GFAP (Jessen et al., 1990) in 7d-PCI nerves. Figure 3A shows similar increases of GFAP signal after injury in WT and ApoD-KO samples, arguing against different numbers of Schwann cells in the ApoD-KO nerves. We then measured the expression of the macrophage gene Emr1/F4-80 by RT-qPCR (Figure 3B), and the expression and tissue distribution of the activated macrophage marker CR3-Cd11b by immunohistochemistry (Figure 3C) and immunoblot (Figure 3D) in injured nerves. The three experimental approaches indicate that macrophage numbers are already increasing at 7d-PCI, and more so in ApoD-KO than in WT nerves. Our previous explorations of late times after injury (14 and 48d-PCI) show that this increase persists and enlarges over time in the absence of ApoD, and that myelin debris accumulates by 14d-PCI (Ganfornina et al., 2010). However, by 7d-PCI, no differences in the extent of myelin clearance was observed, as monitored by immunoblot and immunohistochemistry of Mbp (Figures S1A,B) and by LFB histochemistry (Figure S1C). Thus, the elevated presence of macrophages expressing CR3-Cd11b in ApoD-KO injured nerves contrasts with the normal myelin amounts at this stage of WDR, and suggests a differential effect of ApoD on macrophage recruitment and initiation of phagocytosis.

**Figure 4:**
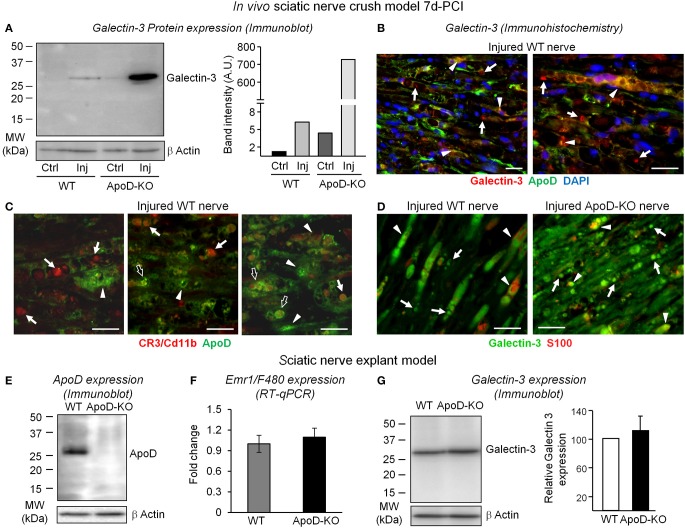
**ApoD-dependent changes in the levels of Galectin-3 expression in nerves 7d-PCI and cultured nerve explants. (A)** Immunoblot analysis of Galectin-3 in the region distal to the lesion of WT and ApoD-KO mice sciatic nerves. The induction of Galectin-3 protein expression by injury is exacerbated in the ApoD-KO nerves at 7d-PCI. Basal expression of Galectin-3 in ApoD-KO nerves is also increased compared to WT nerves. **(B)** Representative micrographs of Galectin-3 and ApoD immunofluorescence experiments in crushed WT nerves 7d-PCI. Arrows point to Galectin-3 labeling in macrophages, and arrowheads indicate co-localization of ApoD and Galectin-3 in SCs and myelin profiles. **(C)** CR3/Cd11b and ApoD labeling in sagittal sections of the injury region of WT nerves 7d-PCI. CR3/Cd11b-positive macrophages do not express ApoD (arrows), which is present in other cells and in myelin profiles (arrowheads). However, some macrophages show partial co-localization with ApoD-positive myelin profiles (open arrows). **(D)** Galectin-3 and S100 labeling in sagittal sections of the injury region of WT and ApoD-KO nerves 7d-PCI. Galectin-3 labeling is more abundant in ApoD-KO nerves, and often co-localizes with S100-positive SCs in the injured nerves. **(E–G)** Analysis of nerve explants cultured for seven days. The graphs represent the average ± *SD* of 3 independent culture experiments. **(E)** Immunoblot analysis of ApoD shows high levels of expression in the WT cultured nerves. **(F)** Emr1/F4-80 mRNA expression does not differ between ApoD-KO and WT nerve explants, indicating similar presence of endogenous macrophages. **(G)** Immunoblot analysis of Galectin-3 shows no significant increment of expression in ApoD-KO compared to WT nerve explants. Protein band intensities were normalized to β-actin. Calibration bars: 25 μm. Statistical differences were assayed by Student's *t*-Test.

We then checked the expression of Galectin-3, a factor involved in signaling macrophages to start myelin phagocytosis, in WT and ApoD-KO nerves (Figure 4). While nerve injury results in over-expression of Galectin-3 protein in the wild type at 7d-PCI, a far greater increase is found in the distal region of crushed nerves in the absence of ApoD (Figure 4A). A number of cells expressing Galectin-3 in distal regions of injured WT nerves express ApoD as well (arrowheads in Figure 4B). They are probably denervated SCs undergoing their phenotypic switch toward repair-promoting phagocytic and macrophage-recruiting cells. We have demonstrated that peritoneal macrophages do not express ApoD (**Figure 6F**). Likewise, macrophages recruited to injured WT nerves do not show ApoD expression (arrows in Figure 4C). However, some tissue macrophages show partial co-localization with ApoD-positive structures (arrows in Figure 4C), which might represent myelin particles that are being phagocytosed (open arrows in Figure 4C). Thus, the Galectin-3-positive cells that do not express ApoD after injury are probably macrophages (arrows in Figure 4B).

The increased Galectin-3 expression and its dependence on ApoD are also clear in immunohistochemistry experiments of WT and ApoD-KO nerves (Figure 4D). Galectin-3-positive cells represent both S100-positive SCs (arrowheads in Figure 4D) and S100-negative macrophages that are being recruited at this time point after injury (arrows in Figure 4D).

#### Resident vs. infiltrated phagocytic cells

These results led us to ask whether infiltrating macrophages are the source of the ApoD-dependent over-expression of Galectin-3. Alternatively, this expression might be due to denervated SCs cells or resident macrophages. To solve this question we performed nerve explant cultures and assayed protein and mRNA expression after 7 days in culture. In this *ex vivo* experimental model all endogenous factors in the nerve are present, with the exception of infiltrating macrophages. This procedure resulted in the expected over-expression of ApoD protein in WT nerves (Figure 4E), but we found no significant ApoD-dependent changes in the macrophage marker Emr1/F480 (Figure 4F) or in Galectin-3 expression (Figure 4G) (representing in this case the activity of resident cells). These results demonstrate that, *in vivo*, bone marrow-derived infiltrated macrophages are the major cell type over-expressing the phagocytosis activator Galectin-3 in ApoD-KO injured nerves. Moreover, they further support that ApoD over-expression in the WT injured nerves originates in nerve resident cells.

#### Myelin breakdown and lipid mediators of inflammation

**Figure 5:**
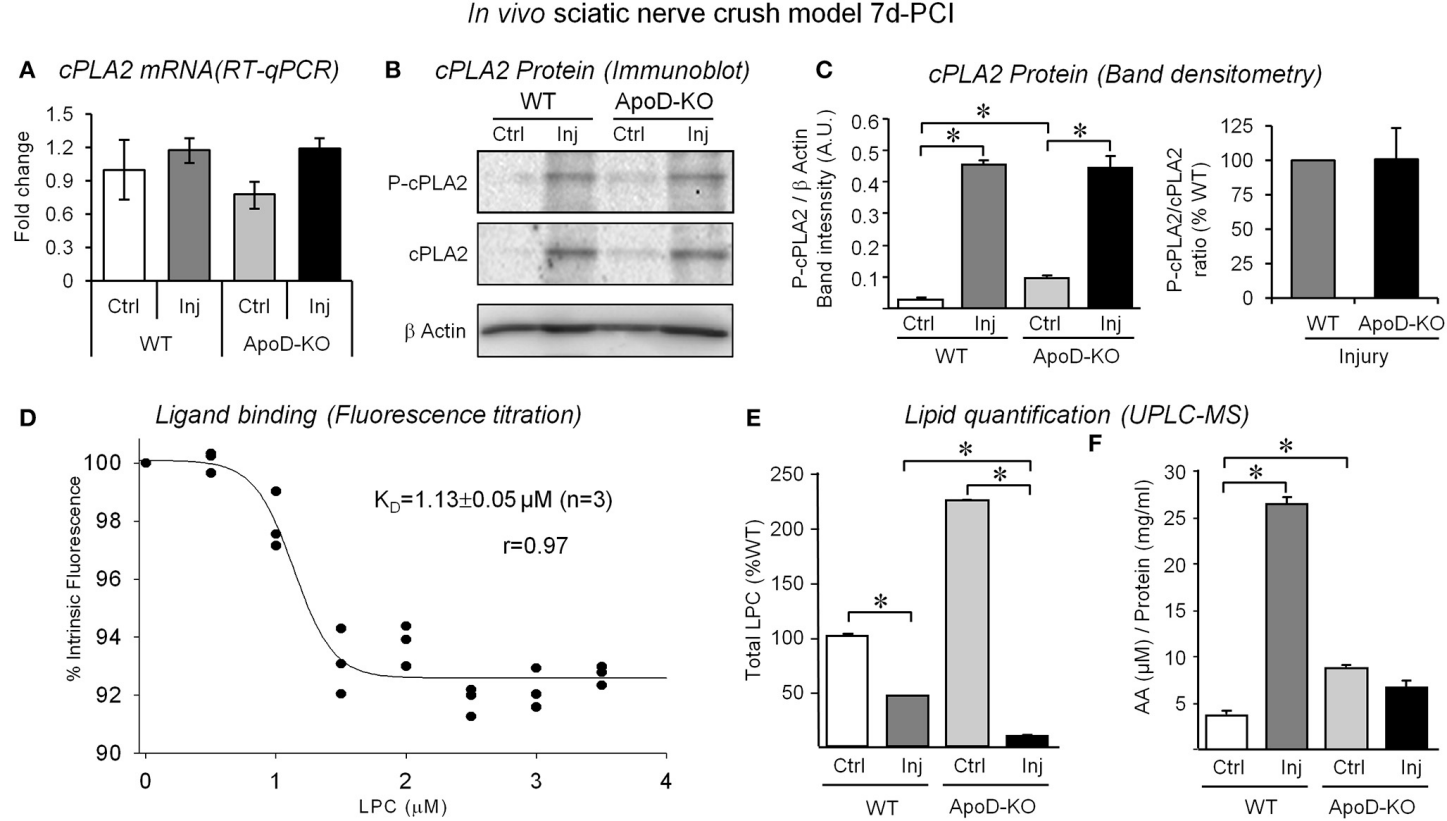
**Analysis of cPLA2 expression, LPC binding to ApoD, and quantification of LPC and free AA in the nerve upon injury. (A)** Quantitative RT-PCR of cPLA_2_ group IVA shows no differences in ApoD-KO nerves 7d-PCI. **(B,C)** Immunoblot analysis of phospho-cPLA_2_ and total cPLA_2_ in intact nerves and in the region distal to the lesion. Neither the induction of cPLA_2_ protein expression upon injury nor its phospho-cPLA_2_/total cPLA_2_ ratio is ApoD-dependent. Protein band intensities were normalized to β-actin. **(D)** Relative fluorescence intensity of hApoD exposed to increasing concentrations of lysophosphatidylcholine (LPC). Dots represent the mean of 3 independent titrations. **(E,F)** Mass spectrometry analyses of LPC **(E)** and free arachidonic acid (AA) **(F)** in intact nerves and in the injury region of WT and ApoD 7d-PCI nerves. Statistical differences were assayed by Mann-Whitney *U*-test **(A)**, and Student's *t*-Test **(C–E)**. Asterisks show statistically significant differences (*P* < 0.05).

Since MCP1 and Tnf are known to stimulate the expression of phospholipases that hydrolyze myelin membrane phospholipids, we could expect alterations of lipase-dependent myelinolysis in the absence of ApoD. We evaluated the expression of the Ca^2+^-dependent cytosolic phospholipase A_2_ alpha (cPLA_2_ group IVA, hereafter referred to as cPLA_2_), since it is known to be expressed by Schwann cells (Lopez-Vales et al., 2008). RT-qPCR and immunoblot analysis of the protein and its more active phosphorylated form are shown (Figures 5A–C). Although no clear differences are seen in mRNA levels with injury or genotype, cPLA_2_ increased protein levels (Figures 5B,C) do reflect the reported increased expression in response to nerve injury (Lopez-Vales et al., 2008), suggesting that the earlier peak of induction of cPLA_2_ has already taken place. Detectable higher levels of phosphorylated cPLA_2_ expression are seen in the control intact ApoD-KO nerve (Figures 5B,C), but no differences in the amounts of the phosphorylated form or in its ratio to the non-phosphorylated form are observed between genotypes upon injury (Figure 5C). Therefore, major effects of ApoD on transcription, translation, or post-translational modification of this particular PLA_2_ upon injury can be discarded.

AA is one of the major products resulting from PLA_2_ myelin cleavage activity, probably due to the preference of PLA_2_for membrane phospholipids with AA, and it contributes to the inflammatory response to injury. ApoD is known to bind AA with high affinity (Morais Cabral et al., 1995; Vogt and Skerra, 2001), and nervous system disorders with altered AA levels concur with ApoD over-expression (reviewed by Dassati et al., 2014). Additionally, PLA_2_ cleavage activity also results in the production of lysophospholipids. Among them, LPC are especially relevant in the injured nerve as myelinolytic agents that contribute to building the cytokine inflammatory network and serve as a signal for macrophage recruitment and activation (Hall and Gregson, 1971; Gregson and Hall, 1973). Binding of LPC to another Lipocalin, α-1-acid glycoprotein, has been reported (Ojala et al., 2006), but it has not been tested for ApoD. We therefore performed *in vitro* ligand-binding assays by tryptophan fluorescence titration of purified human ApoD (Figure 5D). LPC was able to quench intrinsic protein fluorescence in a dose-dependent manner, indicating a successful interaction with a K_D_ of 1.13 μM, similar to other ligand-ApoD interactions (Ruiz et al., 2013). Thus, it is particularly relevant to assay the effect of an ApoD deficiency on AA and LPC levels in the injured nerve.

Using mass spectrometry, we measured the levels of LPC and AA in lipid extracts from the nerve injury site and contralateral control nerves. Three major LPC species were detected in the sciatic nerves: 16:0/OH-PC, 18:1/OH-PC, and 20:4/OH-PC. In the WT nerves, one LPC species (16:0/OH-PC) was slightly increased by injury above basal control levels, whereas levels of 18:1/OH-PC and 20:4/OH-PC are reduced upon injury. After the expected increase of LPC upon injury, probably due to the elevated cPLA_2_ expression and activity, the decrease in free LPCs detected by 7d-PCI could be related to the ongoing processes of both degradation and recycling into new membrane phospholipids. The most prominent effect of the lack of ApoD is the elevation of LPC levels in intact nerves. Also, LPC decrease upon injury without ApoD, further decreasing total levels of LPC (Figure 5E). AA measurements revealed an increase in WT crushed nerves (Figure 5F), possibly related to the induction of cPLA_2_ expression by injury. Interestingly, ApoD-KO intact nerves show higher levels of free AA than WT control nerves. In contrast, no increase of AA is observed upon injury. Thus, ApoD controls the basal and injury-triggered levels of LPC and AA.

In summary, our analysis of WDR in WT and ApoD-KO sciatic nerves at 7d-PCI demonstrates that an extracellular lipid binding protein, the Lipocalin ApoD, influences the cytokine/chemokine inflammatory network, controls the proper activation/recruitment of phagocytic cells that remove myelin debris, and modifies the availability of lipid mediators that are key factors controlling the pace of the response to injury.

### Myelin clearance assays in cultured peritoneal macrophages

Without ApoD, an increased recruitment of activated macrophages is present at 7d-PCI, and a robust expression of the “eat-me” signal Galectin-3 is taking place (Figures 3, 4). Also, the availability of some lipid mediators is modified, with a faster depletion of both LPC and AA. We know that myelin removal is slowed down later on in the absence of ApoD, and this factor ultimately influences the pace of nerve regeneration (Ganfornina et al., 2010). Our next aim was therefore to analyze the process of myelin-macrophage interaction with the working hypothesis that these phenotypes could be explained if ApoD were a key contributor to the regulation of myelin phagocytosis and degradation.

#### Myelin phagocytosis initiation

**Figure 6:**
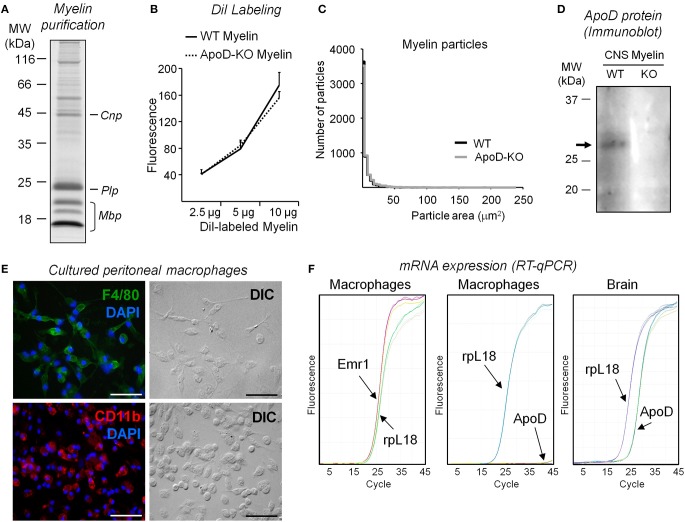
**Characterization and quality controls of myelin extracts and peritoneal macrophage cultures. (A)** SDS-PAGE separation of myelin isolated from brains of WT mice stained with Coomassie blue. **(B)** DiI labeling of myelin assayed by fluorimetry measurements (excitation λ = 545 nm, emission λ = 568 nm). No differences in DiI incorporation are observed in myelin from each mouse genotype. A representative pair of myelin preparations is shown. **(C)** Size distribution of myelin particles in two independent myelin preparations (ten 10× fields/experiment). **(D)** Immunoblot analysis of ApoD in myelin preparations isolated from brains of WT and ApoD-KO mice. The arrow points to the ApoD band. **(E)** Characterization of peritoneal macrophage cultures. The cultured cells express F4-80 and CR3/Cd11b markers, as evidenced by immunofluorescence. Calibration bars: 50 μm. **(F)** ApoD mRNA was not detected by RT-qPCR in peritoneal macrophages, while the macrophage gene Emr1/F4-80 is amplified. Brain mRNA was used to amplify ApoD as a positive control in the same RT-qPCR.

We studied myelin phagocytosis and clearance in cultured peritoneal macrophages to reduce the number of biological variables involved. We used myelin purified from mouse brains (Figures 6A–D) in these experiments because of the scale of source tissue needed. Purified myelin was labeled with DiI (Figure 6B) and particle size distribution was analyzed (Figure 6C) as detailed in the Materials and Methods section. Myelin preparations from WT and ApoD-KO brains show similar distributions of particle size and fluorescence level (normalized to protein amount) (Figures 6B,C). TG-elicited mouse peritoneal macrophages were cultured, and their cell purity was characterized by immunocytochemistry with the macrophage markers Emr1-F4/80 and CD11b (Figure 6E). Emr1-F4/80 gene expression was also confirmed in primary macrophage cultures by RT-qPCR (Figure 6F, left panel). Macrophages lack measurable levels of ApoD mRNA (Figure 6F, middle panel) compared with the normal expression of ApoD in brain (Figure 6F, right panel). We first assessed the process of phagocytosis of macrophages fed with DiI-labeled myelin by examining DiI-positive cells in fluorescence microscopy (Figure S2A). We also studied the time course of myelin phagocytosis by detecting myelin basic protein (Mbp) with immunoblot after feeding TG-elicited mouse peritoneal macrophages with myelin and quantifying Mbp in the cell lysates (Figure S2B). The Mbp signal peaks after 1 h exposure, consistent with Slobodov et al. (2001). We therefore chose a range of 20–60 min of incubation of macrophages with myelin to quantitatively assay the extent of phagocytosis by flow cytometry.

**Figure 7:**
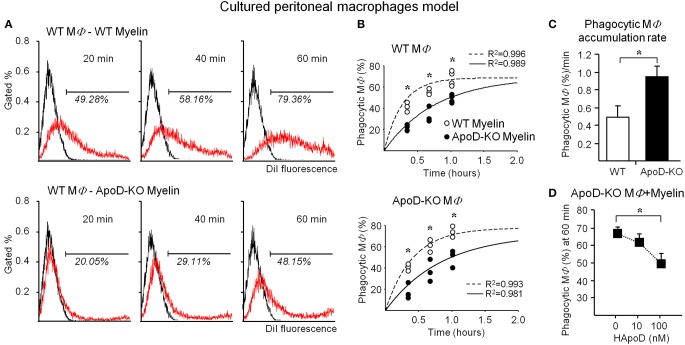
**ApoD alters the phagocytosis of myelin by cultured peritoneal macrophages. (A)** Myelin phagocytosis by TG-elicited macrophages measured with flow cytometry after different times of exposure to myelin of WT or ApoD-KO mouse brains. Histograms of a representative experiment in WT macrophages are shown. The histogram of control macrophages without myelin defines the fluorescence threshold to classify an event as a DiI-positive macrophage. **(B)** Flow cytometry evaluation of myelin phagocytosis by TG-elicited macrophages (WT macrophages, upper panel; KO macrophages, lower panel). Dots in graphs represent average ± SD of 3–4 independent experiments (with 3 macrophage samples per experiment) performed with two myelin preparations/genotype, each purified from 1 to 2 mouse brains. Curve fitting was performed following a single exponential and setting a maximal phagocytosis at 5 h following (Slobodov et al., 2001). ApoD-KO myelin delays macrophage phagocytosis independently of the macrophage genotype. **(C)** Slope of data shown in B for WT and ApoD-KO macrophages, representing the accumulation rate of DiI-positive macrophages. **(D)** The exogenous addition of purified human ApoD to ApoD-KO TG-elicited macrophages rescues the WT levels of myelin phagocytosis in a concentration dependent manner. Statistical differences were assayed by ANOVA followed by a Multiple Comparison Holm-Sidak test **(B)**, and Student's *t*-Test **(C,D)**. Asterisks show statistically significant differences (*P* < 0.05).

Figure 7A shows a representative flow cytometry experiment of macrophage samples after exposure to DiI-labeled myelin. Frequency histograms of macrophage fluorescence without myelin (control) and with DiI-labeled myelin are plotted. The percentage of DiI positive macrophages is significantly smaller upon exposure to ApoD-KO myelin at all times tested. Figure 7B shows the overall results of WT and ApoD-KO macrophages exposed to either WT or ApoD-KO myelin. We fitted our experimental data to a single exponential function with parameters derived from the myelin phagocytosis data analysis previously reported by Slobodov et al. (2001). Our results show that exposure to ApoD-KO myelin clearly results in a delay in the accumulation of DiI-positive macrophages regardless of their genotype (Figure 7B), suggesting an impaired myelin-macrophage recognition or phagocytosis initiation.

The delay is however followed by a 1.8-fold higher accumulation rate of DiI-positive macrophages for ApoD-KO macrophages exposed to ApoD-KO myelin (Figure 7C). When purified human ApoD is exogenously added to ApoD-KO myelin prior to exposure to ApoD-KO macrophages, and phagocytosis is allowed to proceed up to 60 min, the percentage of DiI-positive macrophages is reduced in a dose-dependent manner (Figure 7D).

#### Myelin degradation

**Figure 8:**
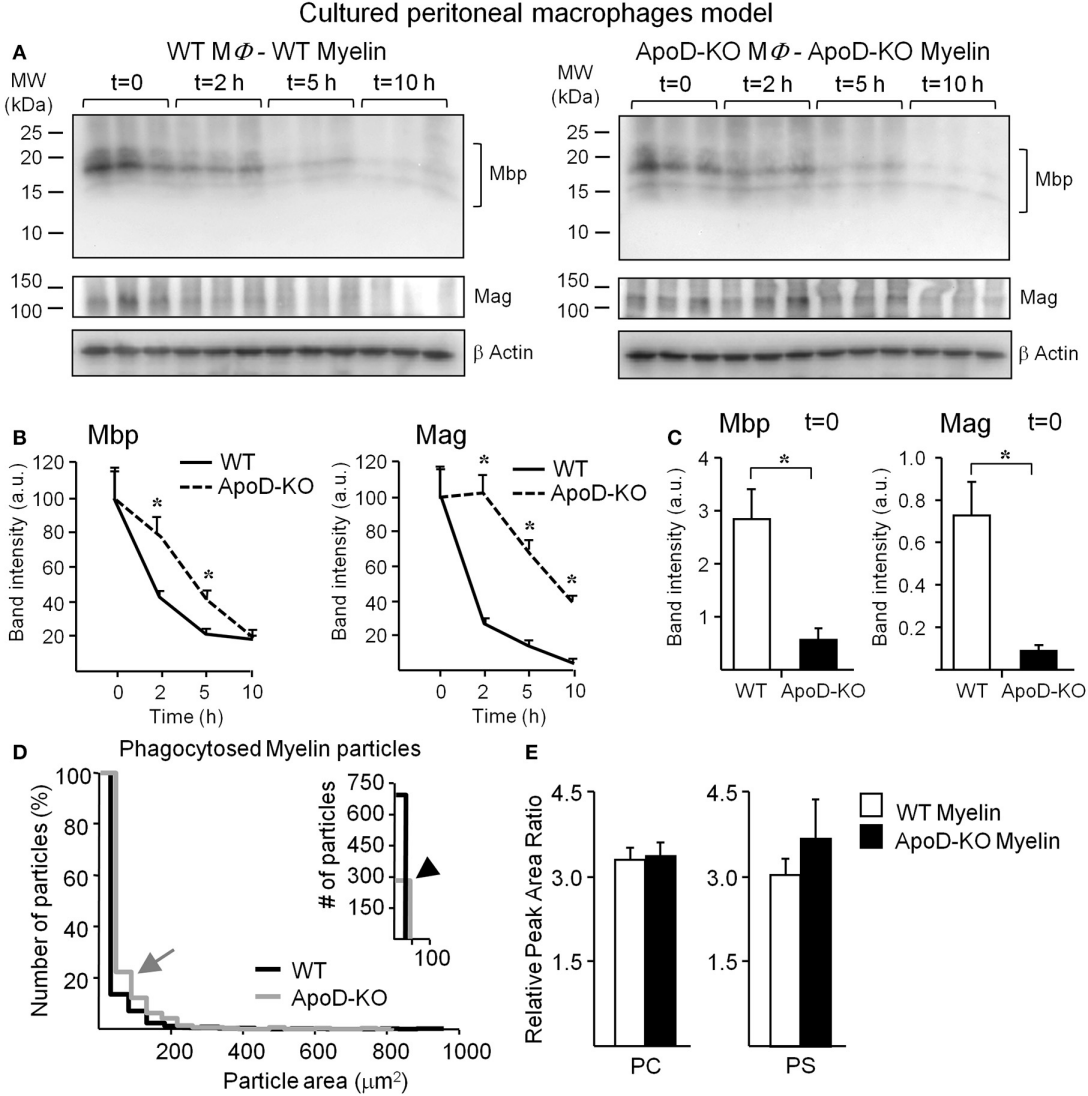
**Myelin degradation by macrophages is delayed in the absence of ApoD. (A)** Degradation assays in cultured macrophages exposed for 60 min to myelin of either WT or ApoD-KO genotype followed by immunoblot analyses of Mbp and Mag at four time points after removal of myelin. **(B)** Band densitometry of Mbp and Mag normalized to β-actin along the four time points explored, and percentually referred to *t* = 0. Average and s.e.m. of three macrophage samples are shown. **(C)** Band densitometry of Mbp and Mag at *t* = 0 normalized to β-actin. **(D)** Size distribution of phagocytosed particles of WT and ApoD-KO myelin referred as a percentage of total number of myelin particles. Right inset shows the number of particles with area <50 μm^2^. Four myelin phagocytosis experiments (6–8 10× fields/experiment) were analyzed for each myelin genotype. **(E)** UPLC-MS analysis of phosphatidylcholines (PC) and phosphatidylserines (PS) in WT and ApoD-KO myelin preparations. The plots represent the average ± *SD* of 6 independent myelin preparations, each extracted from two brains/genotype. Statistical differences were assayed by ANOVA followed by a Multiple Comparison Holm-Sidak test **(B)**, and Student's *t*-Test **(C,E)**. Asterisks show statistically significant differences (*P* < 0.05).

In addition to a myelin-macrophage recognition problem, the differences in the rate of accumulation of myelin-positive macrophages might be due to an altered myelin degradation rate once myelin has been internalized in each macrophage. To test this hypothesis we exposed WT and ApoD-KO macrophages to myelin of their own genotype for 1 h. We then washed away the unbound myelin and monitored myelin degradation by immunoblot of two different myelin proteins, Mbp, and Mag (Figure 8A). Myelin proteins were quantified, normalized with β-actin, and referred to the protein amount at *t* = 0 (Figure 8B). The clearance curves of both myelin proteins show that the absence of ApoD results in a clear delay in myelin degradation. The marked decrease in myelin degradation rate explains why myelin-positive ApoD-KO macrophages accumulate faster over time, even though phagocytosis initiation is delayed. The initial delay is also confirmed with this different methodology since at *t* = 0 (60 min after myelin exposure) the decrease in total Mbp or Mag protein content in macrophages (Figure 8C) agrees with the reduction observed in flow cytometry experiments. An initiation delay combined with inefficient degradation agrees with the delayed myelin clearance observed *in vivo*.

To further confirm the ApoD-dependent degradation deficiency, we measured the area distribution of WT and ApoD-KO myelin particles phagocytosed by TG-activated macrophages (Figure 8D). We found that larger particles of ApoD-KO myelin (gray arrow in Figure 8D) are more represented in the percent distribution of particle sizes present inside macrophages, while the total number of small (<50 μm^2^) WT myelin particles is >2-fold higher than that of ApoD-KO myelin (black arrowhead in inset of Figure 8D). The median particle size was 7.6 μm^2^ in WT and 17.3 μm^2^ in ApoD-KO. These differences in particle size are not present in the original DiI-labeled myelin preparations of either genotype since the area distribution of isolated myelin particles is not statistically different between WT and ApoD-KO myelin preparations (Figure 5C, with median values of 3.3 and 3 μm^2^ in WT and ApoD-KO myelin, respectively). Also, the size comparison between crude and phagocytosed myelin reveal that the differences in ApoD-KO particles found inside macrophages must be due to accumulation of non-degraded material, as the median size of particles inside macrophages is larger than the median size measured in crude myelin.

The anomalous phagocytosis initiation and delayed degradation of myelin particles could be due to differences in myelin composition caused by the absence of ApoD. Using LC/MS/MS, we evaluated in our myelin preparations the amount of the two major phospholipid classes that condition myelin recognition and phagocytosis: phosphatidylcholines (PCs), related to myelinolitic potential, and phosphatidylserines (PSs), that act as “eat me” signals (Hall and Gregson, 1971; Bogie et al., 2013). Figure 8E shows the overall amounts of PCs and PSs in WT and ApoD-KO myelin samples (a list of the detected phospholipids within these classes is shown in Table S1). No differences in the net content of these phospholipids are evidenced, although other compositional differences or redox states of myelin lipids cannot be disregarded as a possibility, given the alterations of LPC and AA observed *in vivo*.

#### Macrophage activation in response to myelin

**Figure 9:**
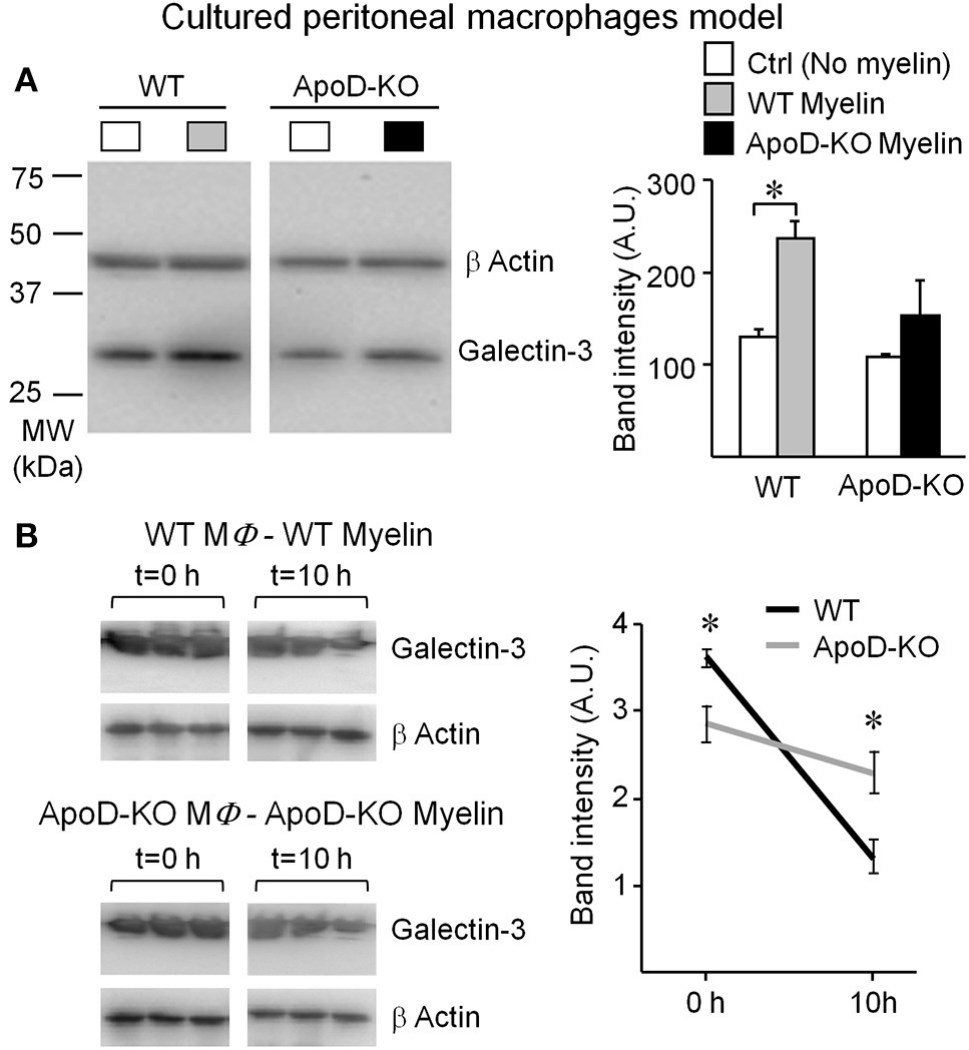
**Macrophage response to myelin is impaired in the absence of ApoD. (A)** Immunoblot analysis of Galectin-3 expression in non-elicited peritoneal macrophages of WT and ApoD-KO mice incubated with myelin. ApoD-KO macrophages do not induce Galectin-3 expression upon myelin exposure. The plot represents average ± *SD* of 3 independent experiments. **(B)** Galectin-3 immunoblot of macrophages exposed for 60 min to myelin of either WT or ApoD-KO genotype, and explored at *t* = 0 and 10 h after removal of myelin. Band densitometry was normalized to β-actin. Statistical differences were assayed by Student's *t*-Test. Asterisks show statistically significant differences (*P* < 0.05).

The results above prompted us to test whether macrophage activation by myelin also depends on ApoD in simplified experimental paradigms to help isolate key variables. We evaluated Galectin-3 protein expression in naïve peritoneal macrophages upon myelin exposure. WT macrophages show increased Galectin-3 expression upon exposure to myelin, but this induction is absent in myelin-fed naïve macrophages from ApoD-KO mice (Figure 9A). When Galectin-3 expression is tested in TG-activated macrophages exposed to myelin of each genotype in the degradation assay (Figure 9B), we observe a decrease of Galectin-3 over time in WT macrophages, as expected for a successful phagocytic activity. However, ApoD-KO macrophages maintain elevated levels of Galectin-3 at the end of the explored time window, supporting the notion of less efficient myelin degradation.

The lack of Galectin-3 response in naïve ApoD-KO macrophages and the lower initial level of Galectin-3 in TG-elicited ApoD-KO macrophages add support to the role of ApoD in the myelin-macrophage initial recognition process. It also indicates that the robust induction of Galectin-3 arising from macrophages infiltrating the injured ApoD-KO nerve *in vivo* (as shown above) must be the combined result of inefficient macrophages accumulating in the nerve.

In summary, the *in vitro* assays performed with cultured peritoneal macrophages reveal both phagocytosis initiation and progression as two distinct processes affected by ApoD. Galectin-3 responses are clearly modulated by ApoD in both isolated macrophages *in vitro* and infiltrating macrophages in the injured nerves *in vivo*. A delayed initiation and inefficient myelin degradation when ApoD is missing explain the results observed *in vivo* in the early phase of WDR explored in this work.

## Discussion

The aim of this work is to gain knowledge into the mechanism by which the Lipocalin ApoD contributes to axon regeneration in peripheral nerves after a crush injury. In our previous work we found that axon regeneration is delayed in ApoD-KO mice sciatic nerves studied 14 and 48d-PCI, probably as a consequence of defects in myelin clearance (Ganfornina et al., 2010). These results open new and early roles for ApoD in controlling phagocytosis by SCs and macrophages that demand further exploration, and that deviate from the initial proposals of potential functions in remyelination. Thus, we have searched for ApoD contributions at 7d-PCI, an early time point of the WDR process when SCs are undergoing a profound phenotypic change (Arthur-Farraj et al., 2012). These denervated SCs and resident macrophages start phagocytosing myelin debris, and their signals recruit blood-derived macrophages (reviewed by Martini et al., 2008; Gaudet et al., 2011; Rotshenker, 2011).

### ApoD expression and function in the intact nerve

A detailed description of the expression and cellular localization of ApoD in the uninjured nerve is needed to fully understand the subsequent response to injury. Our co-localization experiments with immunofluorescence and cell type-specific markers (Figure 1) reveal both myelinating and non-myelinating SCs as the major cell types expressing ApoD in intact PNS nerves. Our results also disfavor a long-standing view of fibroblasts, abundant in the ApoD-negative perineurium, as a major source of the protein (Boyles et al., 1990; Spreyer et al., 1990). Interestingly, not every SC is ApoD-positive in the intact sciatic nerve, in agreement with our previous results in the CNS (Ganfornina et al., 2005), which suggests that particular functional states of SCs benefit from ApoD expression. These results suggest the existence of a regulated transient or intermittent expression of ApoD by SCs in normal conditions. This basal expression, in addition to being required for myelin maintenance (Ganfornina et al., 2010), might contribute to a fast response to an eventual injury. While in this basal mode of expression, ApoD functions as a “break” signal, avoiding the overproduction of pro-inflammatory, pro-myelinolitic, and pro-phagocytosis factors (Tnf, cPLA_2_, LPC, AA, Galectin-3; Figures 2, 4, 5).

### ApoD expression in the injured nerve and its association with myelin

We confirmed the up-regulation by injury of ApoD protein, which can be compared with the results obtained in the rat (Boyles et al., 1990). SCs undergoing the phenotypic switch to a repair-devoted cell are responsible for the ApoD up-regulation upon injury (Figure 1). Seven days after a crush injury these ApoD-positive denervated SCs are phagocytosing myelin particles while attracting blood-derived macrophages to the injury site. Macrophage recruitment is increasing at this time and myelin removal continues until the second week after injury. Then, the nerve environment is cleared of regeneration-inhibitory factors and axons are extending toward their targets (reviewed by Huebner and Strittmatter, 2009). ApoD might have a signaling role for subsequent myelin clearance by SCs and macrophages, since myelin debris present in the 7d-PCI crush site and distal injury region are also ApoD positive (Figure 1). We find ApoD associated with the isolated myelin membrane preparations (Figure 6D), and an association of ApoD with myelin has been previously reported (Suresh et al., 1998; Jahn et al., 2009). This result and the effects of the exogenous addition of ApoD on the phagocytosis assays *in vitro* clearly point to an active role of ApoD, directly or associated with myelin membranes, in modulating the myelin-phagocyte interaction and the progression of phagocytosis.

### ApoD controls pro-inflammation and macrophage recruitment extracellular signals

Upon injury, ApoD controls the cytokine and chemokine network organized by nerve resident cells in a particular and specific manner: only a subset of these extracellular signals is ApoD-dependent (Figure 2). Pro-inflammatory cytokines Il1β and Il6, expressed by SCs and macrophages (Shamash et al., 2002), and by fibroblasts and macrophages (Bolin et al., 1995; Tofaris et al., 2002) respectively, as well as the anti-inflammatory cytokine Il10 expressed by macrophages of the M2 phenotype and involved in tissue repair (Mantovani et al., 2013), are independent of ApoD expression.

The metalloprotease Mmp-9 is induced by Tnf in SCs, regulates the early phases of phagocytic cell activation, and controls the number of resident phagocytic cells in injured nerves (Kobayashi et al., 2008; Chattopadhyay and Shubayev, 2009). Mmp-9 mRNA expression is already decreased by 7d-PCI in our samples, and behaves similarly in both genotypes. These data, and those obtained from nerves cultured *ex vivo* showing levels of resident phagocyte markers unaffected by the ApoD genotype (Figures 4E–G), indicate that the number of resident macrophages and SCs is not influenced by ApoD.

Curiously, the cytokines and chemokines controlled via Toll-like receptor (TLR) signaling are the ones altered by ApoD. The WT transcriptional response of these cytokines and chemokines occurs early (1–2 days) after crush injury, and by 7 days their levels are returning to almost basal levels (Kleinschnitz et al., 2004; Iwatsuki et al., 2013). Again ApoD might exert a “break” function on the post-injury levels of the inflammatory cytokine Tnf, expressed by denervated SCs and macrophages (Shamash et al., 2002), and of the chemokine Ccl2/MCP1 that participates in macrophage recruitment and activation (Tofaris et al., 2002). Without ApoD, an exacerbated and very specific pro-inflammatory response, related to macrophage recruitment and activation, takes place and persists longer. Denervated SCs are still sending recruitment signals to hematogenous macrophages, and these cells are entering the nerve in large numbers according to the expression levels of the macrophage markers (Figures 3B–D) and to our previous results showing increased numbers of macrophages at late WDR stages (Ganfornina et al., 2010).

### ApoD controls local macrophage-activating and pro-phagocytosis factors

Our data demonstrate that ApoD also controls local cues influencing cell activation and phagocytosis, Galectin 3 and Complement receptor-3 (CR3-Cd11b). The β-galactoside binding protein Galectin-3 is expressed and secreted by SCs and macrophages while they clear up axon and myelin debris in injured nerves, and it facilitates the re-programming of the macrophage response toward an anti-inflammatory M2 phenotype (Rotshenker, 2009). The heterogeneous overlap of Galectin-3 and ApoD in SCs of WT injured nerves at 7d-PCI (Figure 4B) reveals that two cellular processes are taking place: myelin scavenging by Galectin-3-positive phagocytic SCs and the launch of remyelination by Galectin-3-negative SCs. In the ApoD-KO injured nerves, the abundance of Galectin-3-positive myelin profiles (Figure 4D) reflects active phagocytosis by both SCs and macrophages, and supports the notion that myelin scavenging is predominant at this time point when ApoD is absent. Therefore, through controlling the expression of Galectin-3, ApoD favors a timely switch to a M2 repair macrophage phenotype. The sciatic nerve explant experiments (Figures 4E–G) demonstrate that these effects of ApoD are exerted mostly on blood-derived infiltrating macrophages.

The activated-macrophage membrane glycoprotein CR3-Cd11b mediates myelin phagocytosis by binding myelin, either directly or through C3bi-dependent opsonization (van der Laan et al., 1996; Reichert et al., 2001; Rotshenker, 2003). ApoD also alters the expression of CR3-Cd11b (Figures 3C,D) further supporting its role as a modulator of macrophage activation.

Thus, upon injury ApoD helps to control the number and activation state of the phagocytic cells recruited to the lesion. If ApoD normally restrains these factors, why is myelin less efficiently cleared up from the lesion when the “break” is removed in the ApoD-KO nerves?

### ApoD promotes myelin-macrophage recognition and myelin degradation

Our analysis in peritoneal macrophages fed with purified myelin reveals two key effects of ApoD: (1) ApoD potentiates macrophage-myelin interaction, since feeding macrophages with ApoD-KO myelin always delays myelin uptake (Figures 7A,B, 8C) and ApoD is needed for appropriate Galectin-3 expression of naïve macrophages upon exposure to myelin (Figure 9A). (2) ApoD promotes efficient myelin degradation, since myelin-positive macrophages accumulate faster without ApoD (Figure 7C), Mbp and Mag proteins remain longer inside macrophages (Figures 8A,B), and macrophages accumulate larger myelin particles (Figure 8D). These effects are in agreement with the persistence of myelin in injured ApoD-KO nerves 48d-PCI (Ganfornina et al., 2010).

We found that adding soluble ApoD to myelin prior to feeding ApoD-KO macrophage cultures results in a rescue of WT phagocytosis parameters, by decreasing the accumulation of myelin-positive macrophages (Figure 7D). We thus propose that ApoD, present in the macrophage environment and able to interact with myelin debris, controls the cell response to myelin at two distinct stages, initiation and progression, and helps to efficiently complete the phagocytosis process.

ApoD is not dispensable for myelin-macrophage recognition and myelin degradation processes *in vivo*: if myelin-macrophage interaction and phagocytosis progression is inefficient (when ApoD is missing), more macrophages and more “eat-me” signals are required.

The myelin-macrophage recognition promoting function of ApoD can be exerted from the extracellular milieu. However, a facilitation of myelin degradation by ApoD would probably require ApoD to function inside the phagocytic cells. We have already shown that ApoD is internalized by glial cells (Bajo-Grañeras et al., 2011). Remarkably, homologs of ApoD in Drosophila promote autophagy flux and protein aggregate clearance in a polyglutamine-based neurodegenerative disease model (del Caño-Espinel et al., unpublished observations). Our data point therefore to ApoD as a multifunctional protein with both, extracellular functions modulating the availability of important signaling lipids (see below), and intracellular functions regulating degradation processes inside cells.

### The lipocalin ApoD controls the fate of lipid mediators influencing inflammatory state, macrophage recruitment and myelin recognition

Phagocytic cells (denervated SCs and macrophages) express upon injury several members of the phospholipase A_2_ family (PLA_2_) in response to MCP1 and Tnf (Murakami et al., 1997). Two important products of PLA_2_ activation in an injured nerve are LPC and AA. In addition to its myelinolitic activity, LPC promotes MCP1 expression by macrophages (Murugesan et al., 2003), thus generating an amplifying loop ultimately conditioning the extent of macrophage infiltration. At the same time, eicosanoids are generated by the oxidation of AA and they contribute to the pro-inflammatory state after injury.

ApoD has been reported to bind AA *in vitro* (Morais Cabral et al., 1995; Vogt and Skerra, 2001) and to reduce oxidized lipids through a conserved Met residue at the entrance of the binding pocket (Bhatia et al., 2011). Measuring free AA in WT nerves by mass spectrometry confirms the expected rise of AA upon injury (Figure 5E), which correlates with the observed increase in cPLA_2_ expression and phosphorylation (Figures 5B,C). Interestingly, a significant increase of AA was also observed in the intact nerve when ApoD is absent. Although a previous report had suggested an ApoD control of free AA, based on AA mobilization after ApoD addition to cell cultures (Thomas et al., 2003), our data demonstrate for the first time that an absence of ApoD increases free AA levels in a live organism. Since cPLA2 alpha is induced and phosphorylated in ApoD-KO nerves similarly to WT nerves, the lower AA levels measured after injury are probably due to other effects of ApoD deficiency. Potential contributing factors could be a lower expression or activity of some other PLA2, faster oxidative catabolism of AA, or increased compensatory reincorporation into phospholipids or triglycerides. Although the elucidation of this mechanism exceeds the scope of this manuscript, our data strongly support a role for ApoD in the control of free AA availability and fate after injury.

The Lipocalin α-1-acid glycoprotein, is known to bind LPC and act as a carrier of extracellular LPC from dying cells to macrophages, contributing as a “presenting agent” to signal the presence of phagocytable material (Ojala et al., 2006). Here we demonstrate that ApoD can bind LPC and control its availability in the nerve tissue. By performing the same molecular role as α-1-acid glycoprotein, ApoD would favor myelin recognition, thus explaining the results obtained with macrophages *in vitro*, and would control macrophage recruitment, by modulating the extent of LPC-triggered MCP1 expression observed *in vivo*.

In summary, the differences in myelin lipid processing could be the major causal factors for the ApoD-KO inflammatory state, increased macrophage infiltration and delayed myelin recognition and degradation.

## Future directions and conclusions

In this work we demonstrate an early role for a lipid binding protein, the Lipocalin ApoD, in several key processes controlling the pace of WDR. The crucial role of denervated SCs in these processes, and the reprogramming of these cells toward a repair-devoted phenotype, are known to be dependent on c-Jun activation after injury (Arthur-Farraj et al., 2012). Since ApoD is a direct downstream target of the JNK pathway in other systems (Hull-Thompson et al., 2009; Bajo-Grañeras et al., 2011), it is reasonable to hypothesize that ApoD is one of the effector genes whose expression is triggered by c-Jun activation, early after injury, as part of the regeneration programming of the transdifferentiated SCs.

Our data demonstrate the existence of ApoD-dependent constitutive differences in the basal inflammatory state, as well as in the dynamics of post-injury phagocyte-myelin interaction and myelin breakdown, where the levels of pro-inflammatory signaling proteins and lipid mediators are altered in the absence of ApoD. We propose a dual molecular mechanism of action that rests on extracellular binding of ApoD to myelin-derived membrane lipids and intracellular promotion of myelin degradation flow by ApoD. After periods of a steady state “idle”-mode of expression in Schwann cells of the intact nerve, ApoD expression is boosted in transdifferentiated Schwann cells after injury, and is present in the myelin debris at the injury site. There, ApoD is able to trigger the appropriate macrophage response and allows for a normal extent and a regular pace of inflammation initiation and termination. This knowledge should help develop ApoD-based pro-regenerative therapies.

### Itemized conclusions

1. Apolipoprotein D (ApoD), a lipid-binding extracellular protein, is expressed in subsets of myelinating and non-myelinating Schwann cells (SCs) in the sciatic nerve. It is associated to myelin membranes both *in vivo* and in myelin preparations. ApoD expression is boosted in the denervated, repair-devoted, SCs as part of the early response to injury.
2. ApoD modulates the cytokine-chemokine network organizing myelin clearance, particularly the response of Tnf and Ccl2/MCP1, both controlled via Toll-like receptor (TLR) signaling.
3. ApoD conditions the cellular response to injury, controlling the infiltration of macrophages and the expression of the pro-phagocytosis molecule Galectin-3, without major alterations in resident macrophages or phagocytic SCs.
4. The control of macrophage behavior *in vivo* is consistent with the myelin phagocytosis assays *in vitro*, which reveals two key effects of ApoD:
  - ApoD potentiates macrophage-myelin interaction. Feeding macrophages with ApoD-KO myelin always delays myelin uptake. ApoD is needed for an appropriate Galectin-3 expression of naïve macrophages upon exposure to myelin.
  - ApoD promotes efficient myelin degradation. Without ApoD, myelin-positive macrophages accumulate faster in the phagocytosis assays, it takes longer to degrade Mbp and Mag proteins, and macrophages accumulate larger myelin particles.
5. ApoD, already known to bind AA, is able to bind lysophosphatidylcholine (LPC), a previously unknown ligand for this Lipocalin.
6. *In vivo*, ApoD controls basal and injury-triggered AA and LPC levels, two major products of phospholipases that operate during myelinolysis and work as signaling lipids in the injured nerve.

In summary, our work reveals novel molecular and cellular mechanisms by which the Lipocalin ApoD benefits nerve injury resolution. It highlights the importance of this lipid binding protein in the availability of extracellular signaling lipids and in the control of macrophage responses to myelin at two distinct stages, initiation and progression.

## Author contributions

Nadia García-Mateo generated the scientific question to pursue based on the lab antecedents. Nadia García-Mateo, Maria D. Ganfornina, and Diego Sanchez contributed to the design of the work. Nadia García-Mateo acquired and analyzed the data with Maria D. Ganfornina and Diego Sanchez helping in both tasks. Olimpio Montero, Miguel A. Gijón, and Robert C. Murphy, designed the work related to lipid measurements, and contributed to the acquisitions and analysis of lipid data. Nadia García-Mateo, Maria D. Ganfornina and Diego Sanchez drafted the manuscript. Olimpio Montero, Miguel A. Gijón and Robert C. Murphy contributed to draft the sections related to lipid measurements and revised the whole manuscript. All authors approved the last version of the manuscript before submission, and are therefore accountable for all aspects of the work.

### Conflict of interest statement

The authors declare that the research was conducted in the absence of any commercial or financial relationships that could be construed as a potential conflict of interest.

## Supplementary material

Figure S1 **The amounts of myelin debris show no differences in WT and ApoD-KO nerves at 7d-PCI. (A)** Immunoblot analysis of Mbp in intact nerves and in the region distal to the injury site at 7d-PCI. Protein band intensity was normalized to β-actin. No difference in the clearing of Mbp-positive myelin debris is detected after injury in ApoD-KO nerves. **(B)** Representative images of Mbp immunohistochemistry in sagittal sections of the lesion region at 7d-PCI. Arrows point at Mbp-positive myelin bodies. **(C)** Myelin staining with Luxol Fast Blue in sagittal sections of the lesion region of crushed nerves at 7d-PCI. Calibration bars: 50 μm. Statistical differences were assayed by Student's *t*-Test.

Figure S2 **Characterization of myelin phagocytosis by cultured peritoneal macrophages. (A)** Fluorescence microscopy of DiI-labeled myelin phagocytosis by TG-elicited peritoneal macrophages after 60 min of co-incubation (representative examples are shown). Calibration bars: 50 (upper panel) and 25 μm (lower panel). **(B)** Myelin phagocytosis time course in TG-elicited peritoneal WT macrophages incubated for up to 24 h with non-labeled myelin from WT mice. The extent of phagocytosis is quantified by the presence of the Mbp myelin protein in the macrophage cell lysates after extensive exchange of the culture medium. Mbp band intensity was normalized to β-actin. The maximum signal was detected at 1 h of co-incubation.
