# Supplementary figures and images for: Schwann cell-derived Apolipoprotein D controls the dynamics of post-injury myelin recognition and degradation
Source: Front Cell Neurosci. 2014 Nov 11;8:374. doi: 10.3389/fncel.2014.00374 (PMC4227524; doi:10.3389/fncel.2014.00374)

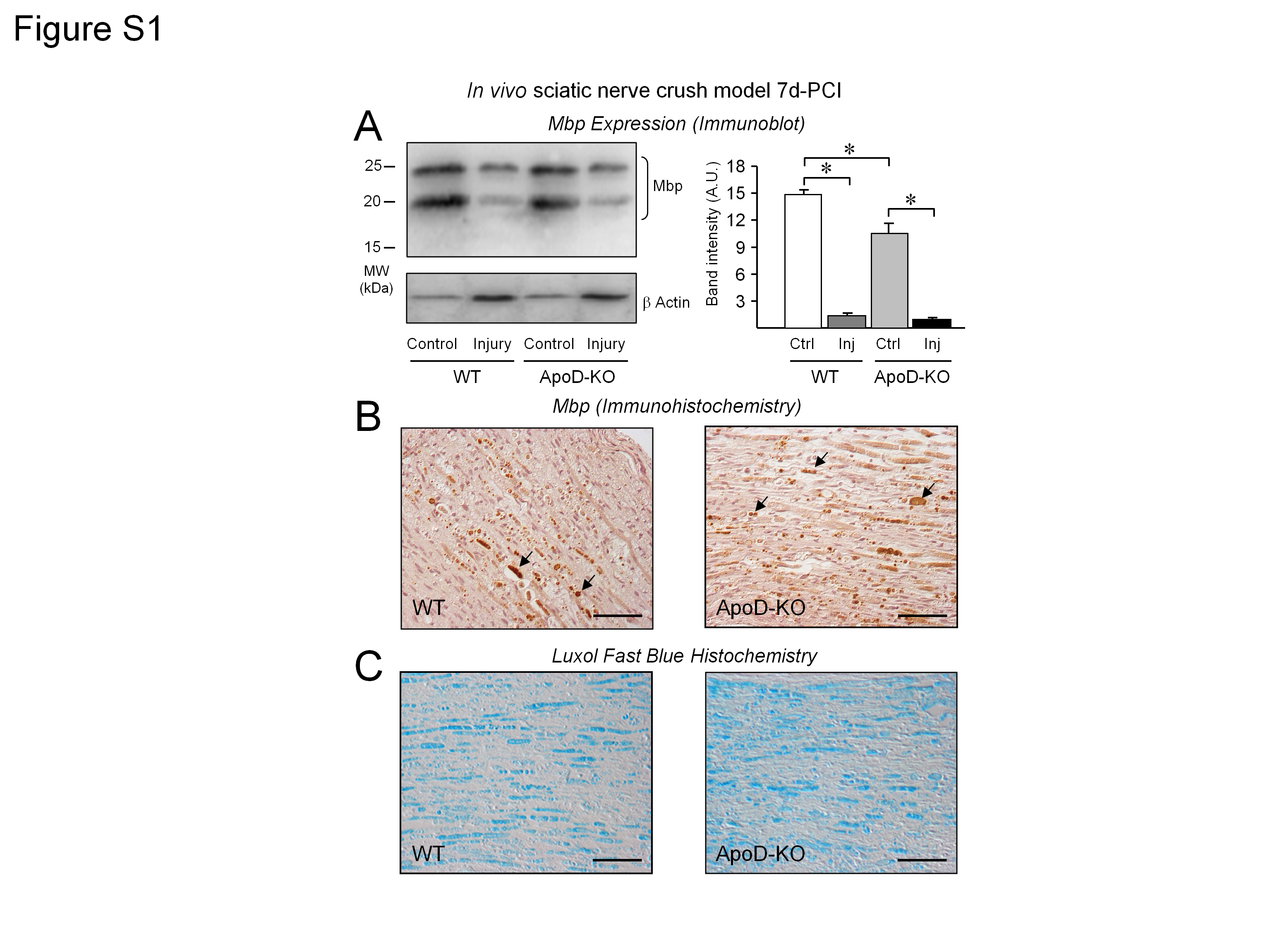

Supplement: Figure S1 — The amounts of myelin debris show no differences in WT and ApoD-KO nerves at 7d-PCI. (A) Immunoblot analysis of Mbp in intact nerves and in the region distal to the injury site at 7d-PCI. Protein band intensity was normalized to β-actin. No difference in the clearing of Mbp-positive myelin debris is detected after injury in ApoD-KO nerves. (B) Representative images of Mbp immunohistochemistry in sagittal sections of the lesion region at 7d-PCI. Arrows point at Mbp-positive myelin bodies. (C) Myelin staining with Luxol Fast Blue in sagittal sections of the lesion region of crushed nerves at 7d-PCI. Calibration bars: 50 μm. Statistical differences were assayed by Student's t-Test. [file Image1.TIF]

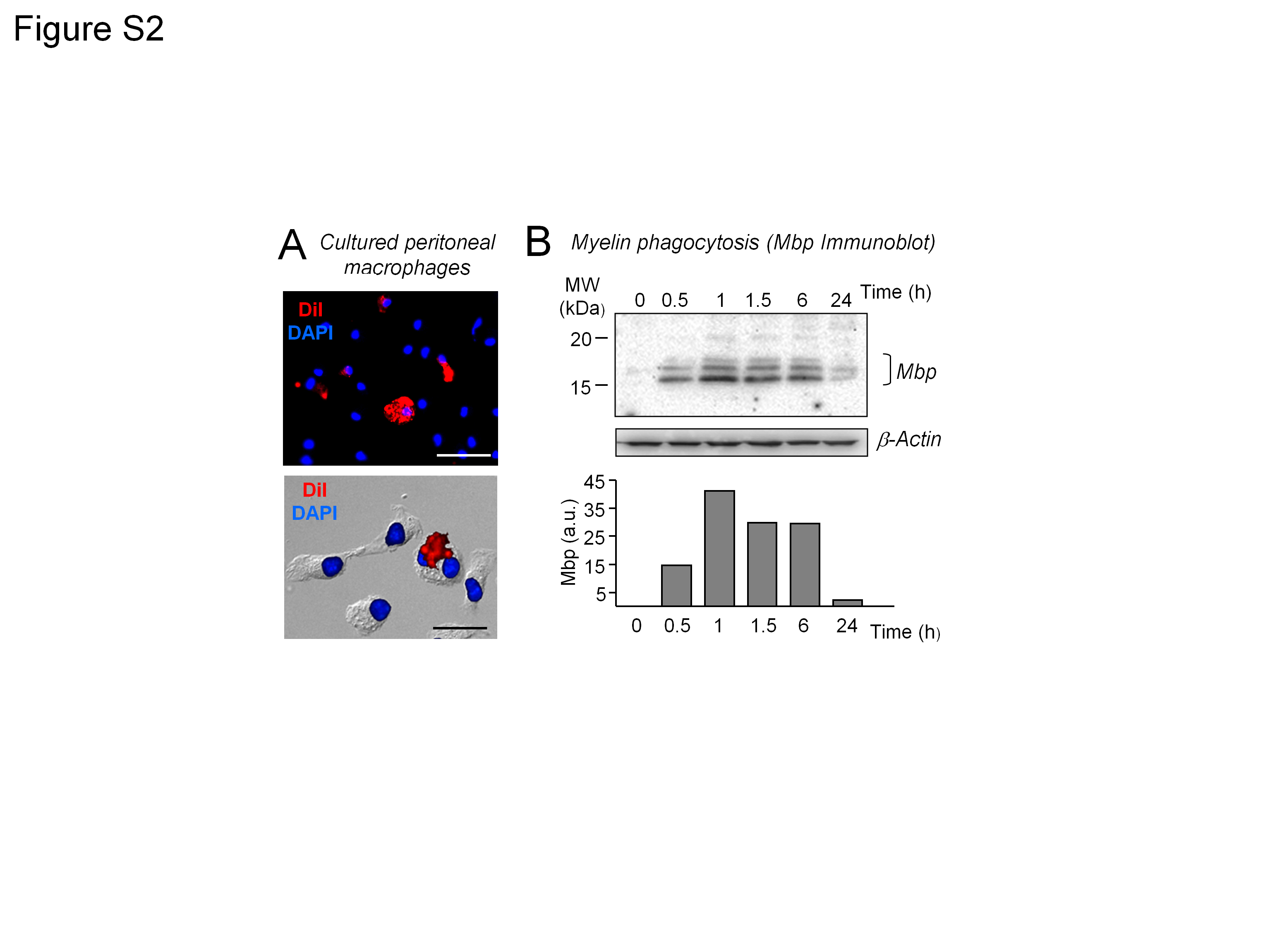

Supplement: Figure S2 — Characterization of myelin phagocytosis by cultured peritoneal macrophages. (A) Fluorescence microscopy of DiI-labeled myelin phagocytosis by TG-elicited peritoneal macrophages after 60 min of co-incubation (representative examples are shown). Calibration bars: 50 (upper panel) and 25 μm (lower panel). (B) Myelin phagocytosis time course in TG-elicited peritoneal WT macrophages incubated for up to 24 h with non-labeled myelin from WT mice. The extent of phagocytosis is quantified by the presence of the Mbp myelin protein in the macrophage cell lysates after extensive exchange of the culture medium. Mbp band intensity was normalized to β-actin. The maximum signal was detected at 1 h of co-incubation. [file Image2.TIF]
